# Patient participation in dialysis care—A qualitative study of patients’ and health professionals’ perspectives

**DOI:** 10.1111/hex.12966

**Published:** 2019-09-27

**Authors:** Liselott Årestedt, Caroline Martinsson, Carina Hjelm, Fredrik Uhlin, Ann Catrine Eldh

**Affiliations:** ^1^ Department of Health and Caring Sciences Faculty of Health and Life Sciences Linnaeus University Kalmar Sweden; ^2^ Department of Medical and Health Sciences Faculty of Medicine and Health Sciences Linköping University Sweden; ^3^ Department of Nephrology University Hospital Linköping Sweden; ^4^ Department of Health Technologies Tallinn University of Technology Tallinn Estonia; ^5^ Department of Public Health and Caring Sciences Uppsala University Uppsala Sweden

**Keywords:** dialysis care, haemodialysis, involvement, patient participation, self‐management

## Abstract

**Background and objective:**

End‐stage renal disease (ESRD) affects a multitude of aspects in the patient's daily life, often entailing their own involvement in various aspects of the treatment. Although patient participation is a core health‐care value, what the concept signifies is not yet fully known. The purpose of this paper is to conceptualize patient participation in dialysis care, depicting patients’ and health‐care professionals’ perspectives.

**Design:**

This explorative study employed qualitative interviews and content analysis.

**Setting and participants:**

Seven focus group discussions engaging 42 key informants were performed, including patients, staff and managers with experience of dialysis care.

**Results:**

In dialysis care, patient participation connotes a sharing of information and knowledge, the learning of and planning of care, including partaking in shared decisions with regards to treatment and management, and being involved in the management of one's own health‐care treatment and/or self‐care activities. Although these attributes were illustrated by all stakeholders, their significance varied: patients suggested that their preferences regarding primary aspects of participation vary, while staff considered patients’ performance of dialysis to be the ultimate form of participation. Further, while patients considered multiple ways to execute participation, staff suggested that aspects such as sharing information were a route to, rather than actual, involvement.

**Conclusions:**

Without a common understanding to denote the idea of patient participation, staff and patients are exposed to a potential deficit in terms of facilitating patient participation in everyday encounters of dialysis treatment. Further studies and means to serve a mutual understanding are needed.

## 1 INTRODUCTION

Patient participation as a core health‐care value has intensified over the last 50 years, supposedly as a result of a collective emphasis on the autonomy of individuals.^1^ While the conceptualization of patient participation initially lacked the patient voice, more recent concept analyses have included elements of what patients define as patient participation.^2^, ^3^, ^4^, ^5^, ^6^ Thus, there are now opportunities to comprehend what the concept connotes for key stakeholders. As a result, clinicians have better options to facilitate the conditions that are necessary from a patient perspective.

Yet, while health‐care professionals and decision‐makers often relate patient participation to decision making,^7^, ^8^, ^9^, ^10^, ^11^ patients suggest that participation includes a wider range of attributes, including being engaged in self‐care, sharing one's experiences of symptoms and treatment with health‐care staff, and being involved in planning and decisions vis‐à‐vis care and treatment.^12^, ^13^, ^14^, ^15^, ^16^ With few exceptions, studies that engage with patients in dialysis care to depict patient participation and how it can be facilitated are lacking.

Patients affected by end‐stage renal disease (ESRD) experience a multitude of disruptions to their daily lives. Primarily, ESRD entails dialysis on a regular basis, often at least three times a week for about 4 hours per session, most often in a hospital or outpatient health‐care service unit if treated with haemodialysis. Patients with ESRD often experience a high symptom burden, both physically and emotionally. Besides dialysis (which is time and energy consuming and often involves travelling), ESRD entails compliance with an altered life, including adaptation to technology alongside particular food and fluid regimens.^17^ Thus, living with ESRD more or less necessitates patient engagement—corresponding to participation as in ‘being involved in activities in daily life’.^18^

To facilitate patient participation, patients and health‐care professionals need a shared understanding of the concept. Besides studies on patient participation vis‐à‐vis self‐care and shared decision making,^15^, ^19^, ^20^ little is known about dialysis patients’ and staff experiences of the concept.

The purpose of this paper is to conceptualize patient participation in dialysis care, depicting patients’ and health‐care professionals’ perspectives.

## 2 METHODS

### 2.1 Design

This explorative study employed qualitative data collection and analysis.^21^

### 2.2 Setting and sample

A region representative of Swedish health care was identified, with seven dialysis units at university, county and local hospitals. All invited units engaged in the study, and written consent was obtained from each unit's department head. Once inclusion was agreed, the manager of each unit was contacted, requesting a time and location for a focus group discussion (FGD). FGDs were suggested over individual interviews to stimulate a comprehensive discussion, informing a conceptualization.^21^ The first‐line managers were asked to identify 2‐3 patients with experience of dialysis due to ESRD, 2‐3 health professionals with experience of facilitating dialysis care and 1‐2 dialysis unit managers. Inclusion criteria applied aimed to secure that all participants had experience of patient participation: patients should have encountered at least five dialysis sessions; staff should have performed dialysis at least for six months; and the managers should be in charge of first‐line issues, including measures for quality of care at unit level.

The people suggested for the FGDs received verbal and written study information, including assurance that their participation was voluntary and based on the individual's own choice. Participants were also guaranteed confidentiality and secure data storage. Individual informed consent was attained in writing from all participants. The FGDs were held from March through May 2018. The study conforms with the World Medical Association's ethical principles,^22^ and ethical approval was obtained by the Regional Ethical Review Board of Linkoping, Sweden (ID 2017/544‐31).

### 2.3 Procedure

The FGDs were held in a separate room at each of the dialysis units, at a time convenient for the informants. The participants were seated as to encourage a mutual conversation, and the introduction prompted the individuals to voice their thoughts and views, by means of their role in dialysis care. All FGDs were guided by an interview guide with open‐ended questions developed for the study, including two main areas: asking the participants (ie patients and staff, including managers) to depict what patient participation is, and what are prerequisites for patient participation (the latter reported separately). Each area included one open, main question: for depicting patient participation, as in this paper, this was raised as ‘can you please depict what patient participation is [to you]?’. No definition or assessment of patient participation was introduced; instead, the FGDs were initiated by the participants’ sovereign reflection on what patient participation connotes (followed by what factors they deem to facilitate and impede patient participation, respectively, reported elsewhere).

The FGDs lasted between 49 and 71 minutes, and were facilitated by a skilled researcher guiding the FGD: for each FGD, there was one researcher facilitating the discussion and a non‐participant observer, both seated to the side of the participants. All seven FGDs included representatives from all three groups of stakeholders; observations confirmed that all participants equally engaged in the discussions. Audio recordings were made and later transcribed verbatim by a trained secretary. An equivalent procedure was maintained between FGDs by following a script for the introduction, the agreed interview guide and two researchers directing the FGDs, either acting as facilitator or non‐participant observer. Both researchers had extensive experience in qualitative interviewing with individuals and groups, including FGDs.

### 2.4 Analysis

Data were analysed with content analysis,^23^ inspired by text interpretation;^24^ the purpose was to appreciate the meaning of patient participation by means of how the stakeholders conceptualize their experience of the phenomenon.

- In the *preparation phase*, an inductive approach was applied: all FGD texts were read and reread several times to grasp the meaning of the text as a whole. The researchers formed separate texts that informed a mutual script, following discussions.
- In the subsequent *structured analysis,* data were considered in relation to 12 attributes conceptualized as patient participation by means of semantics, research findings and including patients’ conceptualizations.^25^ That is, the structured analysis was deductive, employing a contemporary matrix for patient participation, considering both if there were indications that the attribute(s) conveyed what is patient participation in dialysis care, how it is conceptualized in this particular health‐care context, and whether there were similarities and differences as to the voices which related to if one participated as a patient, staff or manager.
- To conclude, a comprehensive understanding was formed, incorporating the initial understanding and the structured analysis. During this final phase, variations between the depictions of patients and health professionals were further illuminated.

The entire analysis was performed separately and collectively, engaging all researchers/authors, with repeated dialogue and reiteration of the transcripts.

## 3 RESULTS

An overview of the demographics of the participants is presented in Table 1.

**Table 1:** Overview of demographics of the participants

	Sex	Age	Profession
Patients	Women 5 Men 10	30‐82 (median 59)	–
Staff	Women 18 Men 0	25‐58 (median 41)	RN or physician
Managers	Women 9 Men 0	38‐63 (median 51)	–

The findings are presented by means of describing the naïve understanding, followed by the structured analysis, and concluding with the comprehensive understanding of patient participation.

### 3.1 Naïve understanding

Patient participation is a complex concept which includes various aspects. Its purpose is to strengthen and promote the patient's health process. In dialysis care, it is preordained for the patient to participate, as the illness requires that the patient takes responsibility. What patient participation signifies to individuals varies between them, but also over time for each individual. Patient participation means taking part in decisions and requiring knowledge transformed into understanding for oneself, the illness and the treatment. It also signifies performing activities related to the treatment of ESRD. Patient participation incorporates a mutual learning relationship between the health‐care professionals (HCPs), and the patient, based on compassion and confidence.

### 3.2 Structured analysis

#### 3.2.1 Being listened to by the health‐care staff

Patient participation means being recognized as a person and signifies respect for one's knowledge and experience as an individual living with a long‐term illness, in this case ESRD. Patient participation is facilitated by the HCPs being willing to listen to and recognize the patient's sharing of their condition and preferences, thus valuing the patient experience. One patient said: ‘*And then I noted that it is actually other things than just partaking in decision making. It is about sharing how you are, and being listened to when telling about it*’ (Patient, Interview 1).

#### 3.2.2 One's knowledge and preferences being respected

Patient participation means that the ideas that one shares are acknowledged, those relating to what may work or not in the dialysis, the prescribed regimen, or regarding one's self‐care and everyday life. Further, patient participation connotes recognizing and learning from others, particularly from fellow patients.

Patient participation can also signify surrendering the health care and associated decisions to the HCPs, once one's preferences have been acknowledged; even if the decision is relinquished to the HCP, a sense of participation occurs, given that one's experience is amalgamated with the professional knowledge and experience of the HCPs, informing the final choice.

Patient: I´ve never been in a situation where someone would say: ‘Do that! You have no say’. I think it has always been a dialogue.

HCP: I think it is about recommending… I can recommend you// So you don´t have to force people, the patient get an understanding of why the nurse or physician suggest something. And that’s participation, you can make a choice. (Interview 2)

#### 3.2.3 Having conditions for mutual communication

Openness and continuity between the patient and the HCP sustains a confident relationship, facilitating patient participation. The relations formed between patients and HCPs in dialysis care differ from other health‐care relations: due to repeated and lengthy interactions, the patient and the HCPs get to know each other well. A genuine yet not intimate relationship is optimal, securing a professional but sincere interaction. Being recognized as an individual, with a life beyond the illness and dialysis, facilitates a sense of being part of one's health‐care team.

Patient: You meet so often and form a relationship, not only as a HCP and a patient. It’s rather on a different level.

HCP: Meanwhile, you talk about so much more. You get to know each other and I think it makes it easier for patients to participate, when you know each other//and you become more open and tell how you feel. (Interview 1)

A sense of mutual trust contributes to patient participation, as the patient can discuss his or her concerns with the HCPs. Sharing the experience of one's illness explains the choices one makes as a patient. Further, participation is sustained by the HCPs’ sharing of their actions, along with explanations about what they suggest and enact. This reciprocity contributes to a wider sense of understanding of one's illness and aids coping in daily life.

#### 3.2.4 Sharing symptoms/issues

For patients, participation connotes telling about one's symptoms and current condition. The HCPs, on the other hand, discuss laboratory results and indicators with the patient in order to facilitate understanding and to encourage patients to communicate any symptoms, suggesting that an understanding of symptoms is required for patient participation.

HCP: Yes, you share the lab results in a way so that they understand the meaning of the numbers.

Patient: Yes, gradually you learn more about both your body and the treatment. (Interview 6)

#### 3.2.5 Having explanations as to symptoms/issues

Participation connotes a sense of recognition of symptoms: for example, when one (as a patient) begins to understand one's laboratory results over time, the comprehension of how the dialysis works can increase, as will the understanding of how this relates to what to eat and drink, or not.

Patient participation means acquiring new knowledge and becoming more interested in one's illness. When the HCP shares information with the patient, his or her conditions for capturing the information are taken into account; for crucial information, various tools, such as illustrations, are applied.

Manager: It is one of the basic principles here; to focus the sense of health.//How can the individual patient participate to a larger extent? Given the conditions. If it is difficult for the patient to read, due to dyslexia, can we use illustrations instead. Then, also persons who not are Swedish speaking, they can be aided by pictures too. It can be easier really, even if you speak Swedish.

Patient: Yes, it helped me, it’s easier to see a picture than to capture a text. (Interview 4)

#### 3.2.6 Getting explanations as to the procedures performed

Although information is deemed a vital attribute for patient participation in dialysis, the content is complex; thus, HCPs gradually share information about procedures, in order to facilitate increasing involvement. To patients, being provided with and processing information is participation, as long as there is a sense of coherence as to what is communicated and how; participation is both to share and to acquire knowledge. Yet, information facilitates participation only if delivered in appropriate portions, recognizing the individual's needs and preferences, including assurances that the information is reiterated when needed.

HCP: I think you can sense it, when you have shared too much, the patient cannot receive more information. Then you can talk more about it the next time//to let it take time, because patients are different and can be affected in various ways‐ for example high uremic toxins or that you just have a tough day. (Interview 5)

#### 3.2.7 Knowing what is planned

The onset of dialysis is a point when patient participation is limited: neither preferences nor shared decisions are applicable, but the patient can only yield to the fact that one's kidney failure requires treatment. Yet, the onset of the need for dialysis can be more or less acute, and patients with a trajectory where the illness has manifested gradually can engage in planning for dialysis. For others, an acute start to dialysis is a speedy decision, irrespective of how much or how little the patient knows. This influences whether the patient understands the plans and actions, and thus feel more or less a participant, or merely endures the treatment at this stage.

Patient: You have to try it before you can decide. Everyone wants to participate, but in different ways// And then you engage more, gradually, when you understand the treatment// but you forget and have to ask many times. And the staff are always willing to explain.

HCP: But then we repeat the information over and over again. (Interview 3)

#### 3.2.8 Taking part in planning of care and treatment

The more settled the dialysis, the more opportunities there are to participate in terms of being involved in the planning of one's care and treatment. This includes, for example, scheduling one's dialyses or trying out different treatment options. One patient said: ‘*Yes, it is a stronger sense of freedom when you can manage the time for the dialysis sessions. If you are on a trip for two days you can change your time for dialysis and then you are not so tied up*’ (Interview 2). Patient participation is facilitated by the HCPs being sensitive to patient needs, although it can be hampered if the HCPs are considered or acting as experts, not recognizing the patient's perspective.

#### 3.2.9 Phrasing personal goals

To formulate personal goals signifies contributing to patient participation, illustrated as setting goals in relation to one's treatment, such as learning to set up the machine for dialysis or abiding fluid restriction, that is goals perceived as being reasonable and within reach. One patient said: ‘*You start with dressing the machine and then you advance gradually. And finally you learn self‐cannulation too. That is good*’. (Interview 3).

#### 3.2.10 Knowing how to manage symptoms

Dialysis care is a process that facilitates patient participation: it is not just a treatment but a way to live. The illness in itself warrants an awareness of what actions can aid a sense of well‐being. As a result, participation signifies knowing how to manage symptoms—some occur at the beginning of the illness and others develop over time. Patients acquire the knowledge by their own experience, and from other patients and the HCPs, aiding an understanding of how things work within one's body and in relation to the dialysis and other treatment options.

Manager: It is part of our way of working. It is not just the nurses who provide the education [session], but you get clues and ideas from other patients too, you keep an eye on who manage their machines and other aspects of the dialysis care themselves. (Interview 4)

#### 3.2.11 Performing care oneself

Current technology allows patients to perform parts of or the entire dialysis themselves. A variety of actions can be performed, representing patient participation, such as trimming the dialysis machine with the necessary devices, and/or self‐cannulation. This is considered to be advanced patient participation by HCPs, requiring self‐confidence and knowledge, the latter acquired by means of attaining information, and engaging in learning opportunities.

Although both patients and staff consider operating the dialysis to be voluntary, HCPs suggest that performing actions in relation to dialysis is a main target. According to patients, choosing to have the staff run the dialysis, at certain times or always, can be an act of patient participation.

However, dialysis is not the sole treatment; patient participation comes in many forms and is associated with the additional management of medications, food and fluids, representing a 24/7 assignment. Thus, kidney failure and dialysis themselves signify patient participation: by gradually gaining control of one's treatment, one can progress in understanding how the symptoms and dialysis interact and counteract.

Manager: We are all people, regardless of an illness or not. We become affected by various things in life. But participation means to be master of the illness instead of that the illness controls you. You have an illness you have to consider, but you can still control your life if you become more involved. (Interview 7)

While the HCPs favour hands‐on patient participation, potential barriers are recognized, particularly in the beginning of dialysis, when patients can fret over operating the dialysis machine. Although the staff are there to serve and aid, and have an unconditional medical liability for the dialysis, actively engaging in parts of or the entire dialysis session is the ultimate patient participation to HCPs.

#### 3.2.12 Managing self‐care

When living with ESRD requiring dialysis, patient participation is constituted by the inevitable self‐care, including managing a restricted nutritional and fluid intake. Although a patient may not engage in the performance of dialysis, having information about food and fluids restrictions and how to comply with them represents a minimum amount of patient participation. HCPs encourage this kind of participation by passing on the information verbally and in writing, reinforcing the patient's understanding of his or her situation. From a patient perspective, participation means knowing what is going on and why. The sense of being one step ahead in one's daily life, in terms of the disease and the treatment, strengthens one's self‐esteem and increases one's self‐confidence, making one a partner in the team to optimize one's health. One HCP said: ‘*Thus, the first question is, how do you want to live your life? How do you want your everday life to be? And how can the illness be integrated into that, that´s the first question to ask, I think’.* (Interview 6).

### 3.3 Comprehensive understanding

In dialysis care, patient participation connotes sharing information and knowledge, learning about and planning care, including partaking in making shared decisions with regard to the treatment and management, and being involved in the management of one's health‐care treatment and/or self‐care activities. Living with ESRD includes an active engagement in managing self‐care around the clock and offers various opportunities to engage in the dialysis, or even running it oneself, either at the dialysis unit or at home. Thus, patients in dialysis partners with the HCPs, through the information and knowledge shared at the clinic, prior to, during or after dialysis.

**Figure 1:**
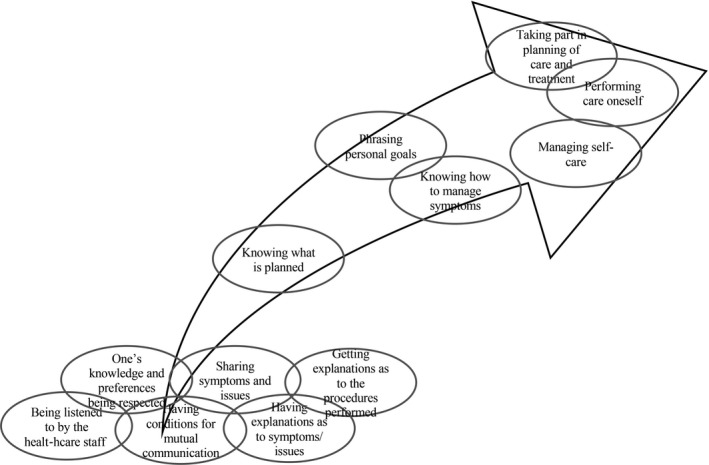
Patient participation according to health‐care professionals’ conceptualizations

Despite the common experience, a variation in the significance of the attributes is noted: to managers and staff, attributes corresponding to a transfer of information are deemed conditional for patient participation, while patient participation is considered as the active involvement of some kind. Thus, knowing (what is planned) conveys a primary level of patient participation, while being involved, knowing how to manage symptoms or phrasing goals (for oneself as a patient) constitute a more advanced level of patient participation. Yet, imparting a hierarchical construction of patient participation, HCPs depict the ultimate patient participation is ‘performing care’ as illustrated in Figure 1.

**Figure 2:**
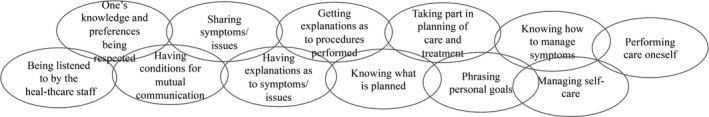
Patient participation according to patients’ conceptualizations

Patients living with ESRD apply the same attributes to patient participation, yet they illustrate that participation connotes different features at different times: sometimes, it means sharing and learning, or actively engaging in running parts of or the complete treatment, while at other times, participation means surrendering the performance of dialysis to the HCPs. What connotes participation can thus change, due to one's sense of well‐ or illbeing and one's current condition, such as level of distress or vigour, although, to patients, the attributes are all linked, as illustrated in Figure 2.

## 4 DISCUSSION

This study highlights that patient participation is a common concept within the dialysis context, for both patients and staff (including managers). Although previously known attributes of patient participation^12^, ^13^, ^14^, ^17^, ^26^ were illustrated in dialysis care, the findings reveal a difference between the significance that each group of stakeholders apply. The HCPs depict the attributes in an order, indicating that patient participation is hierarchical and that communication and information are fundamental to rather than attributes of participation. For the patients, participation instead includes the entire range of attributes, all of which are essential but vary over time. Thus, for example, engaging in a mutual dialogue is not only a route to shared decision making and self‐care, but constitutes participation in itself.^26^ Further, effective collaboration and exchange of information can create mutual understanding between HCPs and patients,^27^ promoting patient participation suitable for dialysis care.

Patients with ESRD gradually become acquainted with their disease, symptoms, self‐care and treatment. They also share their gained knowledge and experiences with staff and fellow patients, thereby participating in and influencing their own care. Consequently, HCPs in dialysis care who recognize the individual's experience and priorities^27^, ^28^, ^29^, ^30^ facilitate not only the patient's active involvement in their care, but also the defining of participation according to their own preferences.

The long‐term relations formed by regular dialysis provide opportunities for the HCPs to support and appreciate the patient's competence and knowledge.^31^ Our findings provide a range of routes to partake and engage, suggesting that staff have multiple opportunities to facilitate patient participation, based on the patients’ willingness, wishes and needs. Yet, staff turnover and a perceived or actual lack of time have been found to hamper patient participation, as will the current lack of basic means to conceptualize patient participation in everyday interactions.^29^ Although staff can promote patient participation by supporting patients to take on the responsibility they elect, forming a team with the patient, engaging in a mutual relationship, and collaborating for the sake of the individual's health and autonomy,^32^ clinical tools to support consensus regarding patient participation in dialysis care are warranted.

Staff lacking competence in promoting that which facilitates participation, and the way the work is organized and performed are suggested barriers for patient participation.^33^ Further, patient‐HCP communication can be hampered by either the staff or the patient considering the patient role as being a passive receiver of information, reduced health literacy and medical jargon used by HCPs, resulting in an ineffective relationship in terms of providing opportunities for patient participation.^34^ Rather, increased knowledge and education promotes patient participation, particularly in terms of self‐care.^35^ While our study verifies the need for HCPs and patients to emphasize patient participation from the individual's preferences,^32^ appropriate means to foster such dialogues may be needed.^25^

This study represents a Swedish health‐care context, corresponding to a legislation where patient participation is highlighted yet not explicitly defined.^36^ Other countries may use a more confined conceptualizing, featuring patient participation as, for example, being engaged in making health‐care decisions.^7^, ^37^ However, as the findings illustrate that patients favour additional attributes when conceptualizing their role, even contexts within a different jurisdiction could consider what the voice of patients may connote, in order to facilitate person‐centred health care.^32^

While the study represents the experiences of both patients and staff, including managers, and includes the voices of different units, individuals with language issues (ie patients who needed an interpreter to participate in a dialogue in Swedish) were excluded. Thus, although the findings justify the similarity of patient participation in dialysis care to that promoted by other patients with long‐term conditions,^12^, ^13^, ^14^, ^26^ further studies investigating, for example, strategies to facilitate patient participation should recognize cultural features of the dialysis context and its clients.

Further, the participants of this study were asked to participate in FGD by a member of staff (ie a unit manager). Had the study meant to assess if or to what extent patient participation occurs, including evaluating the conditions for patient participation, such a recruitment strategy could have imposed a risk of bias. However, with the aim to explore the concept of patient participation in dialysis care, imparting the lived experience of stakeholders, we suggest that participants were likely to speak freely, in particular as facilitated by the common introduction of the study purpose and procedure. Further, the notes confirmed that all participants imparted in the discussions.

During the analysis, attributes conceptualizing patient participation were employed, to further investigate the connotations of patient participation depicted by stakeholders in dialysis care.^23^ Although we identified that all attributes were conveyed, a potential overlap as to how they are conceptualized in this health‐care context was identified. Because the research team members represented a variety of experience of studying concepts, including patient participation, the repeated discussions provided for a critical discourse with regard to the trustworthiness^38^ of the analysis. We aimed for the most liable interpretation^24^ of the concept, originating from the manifest content of the FGDs, yet recognized the potential to further elaborate on the latent content, signifying an interpretation of differences in significance between the stakeholders.^39^ Although this can inform a further understanding, additional studies are probably needed to fully explore attributes of patient participation in dialysis care.

## 5 CONCLUSION

The results showed that in dialysis care, patient participation connotes a sharing of information and knowledge, the learning of and planning of care, including partaking in shared decisions with regard to treatment and management, and being involved in the management of one's own health‐care treatment and/or self‐care activities. Although an increasing understanding of patient participation is at hand, without a common understanding to denote patient participation, staff and patients are exposed to a potential deficit in terms of facilitating patient participation in the everyday encounters of dialysis. Further studies and means to serve a mutual understanding are warranted.

## CONFLICT OF INTEREST

The authors declare that they have no conflicts of interest.

## AUTHORS’ CONTRIBUTIONS

The study was designed by ACE, in collaboration with LÅ, CH and FU. Data were collected by LÅ, CM and ACE. All authors contributed to the analysis and drafting the manuscript. The final version is agreed by all authors.

## ETHICAL APPROVAL

The study conforms with the World Medical Association's ethical principles and was approved by the Regional Ethical Review Board of Linköping, Sweden (ID 2017/544‐31).

## Data Availability

The data that support the findings of this study are available on request from the corresponding author. The data are not publicly available due to privacy or ethical restrictions.
